# Responding to Elder Abuse When Victims Oppose Legal Intervention: Insights from the Front Lines

**DOI:** 10.1093/geroni/igaf122.227

**Published:** 2025-12-31

**Authors:** Catheryn Koss

**Affiliations:** California State University, Sacramento, Sacramento, California, United States

## Abstract

Elder abuse cases present unique challenges when victims are reluctant to prosecute their abusers or take other legal action, often due to familial ties. This qualitative study examines insights from over 20 professionals, including prosecutors, Adult Protective Services (APS) workers, and private attorneys, who work directly with elder abuse victims. Several key themes were identified across the in-depth interviews that highlight the complexities of balancing legal imperatives with victim-centered approaches. One theme is asynchronous time, where the legal system moves too quickly when victims need more time and too slowly when urgent action is required. Another is the importance of an ally, emphasizing the need for a trusted advocate to support victims. Participants also stressed active and open listening, which helps victims share their stories, reduce shame, and provide prosecutors with valuable context for decision-making. A tension emerged around division of labor and continuity of support—while multiple professionals must be involved, victims also need a consistent contact person for trust and stability. Keeping victims informed is also critical, yet professionals disagreed on how much influence victims should have over prosecution decisions. Some advocated for incorporating victims’ concerns into decisions about legal responses, including sentencing recommendations, while others emphasized broader criminal justice priorities and the importance of protecting victims and others from harm. This presentation will explore these and other themes and discuss strategies for navigating elder abuse cases when victims resist or oppose legal action. Attendees will gain insights into improving responses and fostering victim engagement in the legal process.

